# Gene expression changes reflect clinical response in a placebo-controlled randomized trial of abatacept in patients with diffuse cutaneous systemic sclerosis

**DOI:** 10.1186/s13075-015-0669-3

**Published:** 2015-06-13

**Authors:** Eliza F. Chakravarty, Viktor Martyanov, David Fiorentino, Tammara A. Wood, David James Haddon, Justin Ansel Jarrell, Paul J. Utz, Mark C. Genovese, Michael L. Whitfield, Lorinda Chung

**Affiliations:** Arthritis and Clinical Immunology, Oklahoma Medical Research Foundation, Oklahoma City, OK USA; Department of Genetics, Geisel School of Medicine at Dartmouth, Hanover, NH USA; Department of Dermatology, Stanford University School of Medicine, Stanford, CA USA; Division of Immunology and Rheumatology, Stanford University School of Medicine, Stanford, CA USA; Palo Alto VA Health Care System, Palo Alto, CA USA

## Abstract

**Introduction:**

Systemic sclerosis is an autoimmune disease characterized by inflammation and fibrosis of the skin and internal organs. We sought to assess the clinical and molecular effects associated with response to intravenous abatacept in patients with diffuse cutaneous systemic.

**Methods:**

Adult diffuse cutaneous systemic sclerosis patients were randomized in a 2:1 double-blinded fashion to receive abatacept or placebo over 24 weeks. Primary outcomes were safety and the change in modified Rodnan Skin Score (mRSS) at week 24 compared with baseline. Improvers were defined as patients with a decrease in mRSS of ≥30 % post-treatment compared to baseline. Skin biopsies were obtained for differential gene expression and pathway enrichment analyses and intrinsic gene expression subset assignment.

**Results:**

Ten subjects were randomized to abatacept (n = 7) or placebo (n = 3). Disease duration from first non-Raynaud’s symptom was significantly longer (8.8 ± 3.8 years vs. 2.4 ± 1.6 years, *p* = 0.004) and median mRSS was higher (30 vs. 22, *p* = 0.05) in the placebo compared to abatacept group. Adverse events were similar in the two groups. Five out of seven patients (71 %) randomized to abatacept and one out of three patients (33 %) randomized to placebo experienced ≥30 % improvement in skin score. Subjects receiving abatacept showed a trend toward improvement in mRSS at week 24 (−8.6 ± 7.5, *p* = 0.0625) while those in the placebo group did not (−2.3 ± 15, *p* = 0.75). After adjusting for disease duration, mRSS significantly improved in the abatacept compared with the placebo group (abatacept vs. placebo mRSS decrease estimate −9.8, 95 % confidence interval −16.7 to −3.0, *p* = 0.0114). In the abatacept group, the patients in the inflammatory intrinsic subset showed a trend toward greater improvement in skin score at 24 weeks compared with the patients in the normal-like intrinsic subset (−13.5 ± 3.1 vs. −4.5 ± 6.4, *p* = 0.067). Abatacept resulted in decreased CD28 co-stimulatory gene expression in improvers consistent with its mechanism of action. Improvers mapped to the inflammatory intrinsic subset and showed decreased gene expression in inflammatory pathways, while non-improver and placebos showed stable or reverse gene expression over 24 weeks.

**Conclusions:**

Clinical improvement following abatacept therapy was associated with modulation of inflammatory pathways in skin.

**Trial registration:**

ClinicalTrials.gov NCT00442611. Registered 1 March 2007.

**Electronic supplementary material:**

The online version of this article (doi:10.1186/s13075-015-0669-3) contains supplementary material, which is available to authorized users.

## Introduction

Systemic sclerosis (SSc) is an autoimmune connective tissue disease characterized by inflammation and fibrosis of the skin and internal organs, widespread vascular damage, and autoantibody production. Patients with diffuse cutaneous SSc (dcSSc) have extensive fibrosis of the skin, and suffer significant morbidity related to skin tightening including pain, pruritus, and the development of contractures and tendon friction rubs [1].

Although the etiology of SSc remains unknown, several observations support the role of activated T cells in disease pathogenesis. Skin biopsies obtained from SSc patients early in their disease demonstrate a perivascular, mononuclear cell infiltrate comprised of T cells and macrophages [2, 3]. T cell activation is a prominent feature in SSc, as demonstrated by the presence of increased numbers of T cells bearing activation markers, such as interleukin (IL)-2 receptor [4], as well as elevated levels of cytokines such as IL-2, IL-4, IL-6, and IL-17 in the peripheral blood of patients [5–8].

Abatacept (Orencia, Bristol-Myers Squibb, New York, NY, USA) is a soluble fusion protein that consists of the extracellular domain of human cytotoxic T lymphocyte-associated antigen 4 linked to the modified Fc portion of human immunoglobulin G1. Abatacept inhibits T cell activation by binding to CD80 and CD86, thereby blocking interaction with CD28. We conducted a pilot study to assess the safety, tolerability, potential efficacy, and molecular effects of intravenous (IV) abatacept in patients with dcSSc based on the analysis of clinical and gene expression data.

## Methods

### Study protocol

The study is registered with ClinicalTrials.gov, NCT00442611. The Institutional Review Board of Stanford University approved the study prior to its initiation. The study was conducted according to Declaration of Helsinki Principles. All participants provided written informed consent. Study enrollment occurred from May 2008 through November 2010. Eligible subjects were ≥18 years old with a diagnosis of dcSSc. Subjects must have had no symptoms suggestive of renal crisis within 6 months of screening; forced vital capacity (FVC) >49 % and diffusing capacity of the lung for carbon monoxide (DLCO) >39 % predicted, absence of pulmonary hypertension, congestive heart failure, or symptomatic coronary artery disease. Immunomodulatory therapy had to be discontinued at least 90 days prior to randomization, but prednisone ≤10 mg daily was permitted if the dose was stable for at least 28 days prior to randomization. Exclusion criteria included a diagnosis of limited cutaneous SSc, eosinophilic fasciitis, eosinophilia myalgia syndrome, other overlap autoimmune syndromes, or concurrent diagnosis with another definable connective tissue disease, or a known history of any chronic infections.

### Intervention and study assessments

Subjects were randomized 2:1 to receive abatacept dosed according to weight (500 mg/dose for subjects weighing <60 kg; 750 mg/dose for those weighing 60–100 kg, and 1,000 mg/dose for those weighing >100 kg) or matching placebo by intravenous infusion. All other concomitant medications, including treatment for Raynaud’s phenomenon, gastroesophageal reflux disease, non-steroidal anti-inflammatory drugs and prednisone at ≤10 mg daily were continued at stable doses throughout the study period. Subjects were dosed on days 1, 15, 29, and every 28 days for a total of seven doses through day 141. Final study visit for safety and efficacy assessments was day 169 (week 24). At each study visit, subjects underwent physical examination including vital signs and modified Rodnan skin score (mRSS) [9], and laboratory assessment including complete blood count, comprehensive metabolic panel, urinalysis, and erythrocyte sedimentation rate (ESR). Patient global assessment of disease activity and pain by 10-point visual analogue scale (VAS), and physical function assessed by the Scleroderma Health Assessment Questionnaire-Disability Index (HAQ-DI) were collected at each visit as was physician global assessment by VAS. Pulmonary function tests were performed at baseline and week 24. Skin biopsies were obtained from the forearm 10 cm distal to the olecranon at baseline and repeated within 1 cm from the initial biopsy site at week 24 in a subset of patients. Skin biopsy samples were frozen in liquid nitrogen and subsequently thawed at −20 °C in RNAlater-ICE (Ambion, Life Technologies, Grand Island, NY, USA) and RNA prepared for analysis of genome-wide gene expression.

### Masking

Both patients and investigators were blinded to the treatment allocation. The same efficacy assessor (LC) performed all skin scores and physician global assessments at baseline and week 24, and remained blinded to safety assessments throughout the study.

### Outcomes

Safety was the primary outcome, comparing adverse events (AEs) and serious AEs in the abatacept and placebo groups. The primary efficacy endpoint was the change in mRSS at week 24 compared to baseline. Secondary efficacy endpoints included changes in Scleroderma HAQ-DI, patient and physician global assessments, and pulmonary function at 24 weeks compared to baseline.

### DNA microarray hybridization and data processing

Tissue samples were processed and microarray data were normalized and filtered as previously described [10, 11]. cRNA was hybridized to Agilent (Santa Clara, CA, USA) SurePrint G3 Human Gene Expression 8x60K Microarrays (G4851A). Agilent Feature Extraction Image Analysis Software (Version 10.7.3) was used to extract data from raw microarray image files. Probes with >20 % missing data were excluded resulting in 41,589 probes that passed the filtering criteria. The probes were median-centered across all arrays. The gene expression data are available from NCBI GEO at accession number GSE66321.

### Differential gene expression analysis

Missing values in microarray data were imputed using GenePattern [12] module ImputeMissingValuesKNN; 41,589 probes were collapsed to 21,982 gene symbols via GenePattern module CollapseDataset using annotation file for Agilent SurePrint G3 Human GE v2 8x60K Microarray. Genes differentially expressed between two phenotypic classes of interest (e.g., between baseline and post-treatment improver samples) were identified using GenePattern module ComparativeMarkerSelection [13]. Expression data for significant genes (*p* < 0.05) were clustered in Cluster 3.0 [14] and visualized in TreeView [15]. Differentially expressed gene signatures were analyzed for functional enrichment via g:Profiler [16] and were annotated with significantly enriched functional terms (false discovery rate (FDR) <5 %).

### Intrinsic subset assignment

Gene expression data were analyzed for all samples in this study (including abatacept- and placebo-treated patients) as well as four healthy control samples previously analyzed on the same DNA microarray platform. The inclusion of healthy controls was necessary to provide the proper distribution of gene expression data for intrinsic subset assignment. The 26,251 probes from the Agilent 8x60K platform were collapsed to 16,214 unique gene symbols. The 995 intrinsic probes from Milano et al. ([11]; Agilent 4x44K platform) were collapsed to 793 unique gene symbols. Of these 793 unique intrinsic genes from [11], 645 (~81.3 %) were also present in the abatacept dataset and were used in the cluster analysis.

In order to formally assign each sample to the intrinsic gene expression subset, we performed a correlation of centroids for the 645 intrinsic genes between the 20 samples in this study and the reference dataset of [11]. Centroids were calculated for the fibroproliferative, inflammatory and normal-like groups; the limited subgroup was excluded since no patients with limited SSc were included in the abatacept study. The gene expression centroid was created by averaging the gene expression data for all 645 genes across all samples assigned to that intrinsic subset in [11]. We then calculated Spearman correlation statistics (correlation coefficients and *p*-values) between each abatacept sample and three intrinsic subset centroids. We made the intrinsic subset call based on the highest Spearman correlation coefficient and the lowest *p*-value.

### Differential pathway expression analysis

Gene expression data were analyzed using Gene Set Enrichment Analysis (GSEA) [17, 18] module in GenePattern. GSEA was run versus canonical pathway gene sets curated from several databases, including Kyoto Encyclopedia of Genes and Genomes (KEGG) [19] and Reactome [20]. To identify pathways differentially expressed on a single sample level, microarray data were analyzed via ssGSEA (single-sample Gene Set Enrichment Analysis) [21] using ssGSEAProjection module from GenePattern. Significant pathways (FDR <10 %) were clustered and visualized as described above.

### Statistical considerations

The sample size of 10 patients was pre-determined based on the amount of support, drug, and placebo that was provided to conduct this investigator-initiated study. Last observation carried forward analyses were employed. Comparisons between groups were analyzed using Fisher’s exact tests, *t*-tests, and chi-squared analysis where appropriate. mRSS scores were compared using Wilcoxon signed rank test and mixed models to account for repeated measures over eight visit times for each patient and adjusted for disease duration. Statistical analyses were performed and plots were constructed via GraphPad (La Jolla, CA, USA) Prism Windows 6.05.

## Results

### Participant flow and assignment

Twelve subjects were assessed for eligibility and two patients were excluded because IV access could not be obtained. The 10 remaining subjects were randomized in a 2:1 double-blinded fashion to receive IV abatacept (n = 7) or placebo (n = 3). During the follow-up, one patient randomized to placebo withdrew because of an infected digital ulcer. One patient randomized to abatacept declined to provide skin biopsies. Therefore, biopsies for gene expression analyses were available for eight of ten study subjects, including two of the three patients in the placebo group and six of the seven patients in the abatacept group. Five of these six abatacept-treated patients were classified as improvers, defined as a decrease in mRSS of ≥30 % post-treatment compared to baseline.

### Patient population

The mean age was 42.4 ± 12.2 years and 80 % were female, 60 % Caucasian, and 60 % Scl-70 positive. Duration of disease from first non-Raynaud’s symptom was significantly longer (8.8 ± 3.8 years vs. 2.4 ± 1.6 years, *p* = 0.004) and median mRSS higher (30 vs. 22, *p* = 0.05) in subjects receiving placebo compared to abatacept (Table 1).

**Table 1:** Baseline patient characteristics

Variable	Abatacept	Placebo	*p*-value
	n = 7	n = 3	
Age (year)	39.8 ± 11.4	48.6 ± 13.9	0.32
Female (n, %)	5 (71.4)	3 (100)	1
Caucasian (n, %)	4 (57.1)	2 (66.7)	1
Disease duration 1^st^ Raynaud’s (year)	3.9 ± 3.4	9.2 ± 3.2	0.05
Disease duration 1^st^ non-Raynaud’s (year)	2.4 ± 1.6	8.8 ± 3.8	0.004
Scl-70+ (n, %)	4 (57)	2 (66.7)	1
mRSS, median (range)	22 (16–35)	30 (27–33)	0.05
HAQ-DI	0.6 ± 0.8	1.5 ± 1.1	0.18
Physician global VAS	37.6 ± 13.8	56.3 ± 5.5	0.57
Patient global VAS	53 ± 35.8	61.7 ± 44.8	0.75
Patient pain VAS	42.7 ± 35.3	53 ± 47.8	0.71
ESR (mm/hour)	13.7 ± 15.8	31 ± 18.5	0.17
FVC (% predicted)	77.3 ± 19	73.3 ± 27.6	0.79
DLCO (% predicted)	87 ± 17.5	80.3 ± 24	0.65

Values are mean ± SD unless otherwise indicated. *mRSS* modified Rodnan skin score, *DLCO* diffusing capacity of the lung for carbon monoxide, *ESR* erythrocyte sedimentation rate, *FVC* forced vital capacity, *HAQ-DI* Health Assessment Questionnaire Disability Index, *VAS* visual analogue scale

### Follow-up analysis: safety outcomes

Overall, abatacept was well tolerated and AEs were similar between groups with seven reported in each treatment group (Table 2). The most common AEs were infections. One patient, randomized to placebo, developed an infection in a pre-existing digital ulceration of the toe that led to premature withdrawal after the day 114 (16 week) visit. Mild pruritus was noted by 2/7 subjects randomized to abatacept. Only one serious AE occurred in a patient in the abatacept arm who was hospitalized for an episode of supraventricular tachycardia, for which he had a history prior to study enrollment. The AE was felt to be unrelated to the study drug and the subject completed the study.

**Table 2:** Safety and efficacy^a^ outcomes

Variable	Abatacept	Placebo	*p*-value
	n = 7	n = 3	
Adverse events	7	7	1
Serious adverse events	1	0	
Infections^b^	2	4	
Pruritus	2	0	
Lower extremity edema	0	1	
Headache	1	0	
Dry mouth	0	1	
Nausea	0	1	
Fever	1	0	
Absolute change in mRSS, abatacept	−8.6 ± 7.5	–	0.0625
Absolute change in mRSS, placebo	–	−2.3 ± 15	0.75
Change in HAQ-DI	−0.04 ± 0.24	0.25 ± 0.25	0.16
Change in physician global VAS	−11.9 ± 18.1	−17.3 ± 23.2	0.048
Change in patient global VAS	−8 ± 7.6	−2.7 ± 6.7	0.023
Change in patient pain VAS	−11.4 ± 8.3	−15 ± 25.1	0.18
Change in ESR	−6 ± 7.0	1.7 ± 7.6	0.37
Change in FVC % predicted	1.3 ± 8.5	0.3 ± 8.5	0.72
Change in DLCO % predicted	2.0 ± 6.3	−7.4 ± 10.7	0.84

Values are mean ± SD. ^a^Efficacy outcomes are based on comparing week 24 to baseline. ^b^Infections in the abatacept group included two upper respiratory tract infections, and in the placebo group one of each of the following: upper respiratory tract infection, urinary tract infection, hordeolum, and infected toe digital ulcer. *DLCO* diffusing capacity of the lung for carbon monoxide, *ESR* erythrocyte sedimentation rate, *FVC* forced vital capacity, *HAQ-DI* Health Assessment Questionnaire Disability Index, *mRSS* modified Rodnan skin score, *VAS* visual analogue scale

### Follow-up analysis: efficacy outcomes

Subjects receiving abatacept showed a trend toward improvement in mRSS at week 24 (−8.6 ± 7.5, *p* = 0.0625) while those in the placebo group did not (−2.3 ± 15, *p* = 0.75) (Table 2). After accounting for disease duration and repeated measures over eight visit times, the difference between groups was statistically significant (abatacept vs. placebo mRSS decrease estimate −9.8, 95 % confidence interval −16.7 to −3.0, *p* = 0.0114) (Table 3). Five of seven patients (71 %) randomized to abatacept and one of three patients (33 %) randomized to placebo experienced a ≥30 % improvement in skin score (*p* = 0.5). The effects on global VAS were in opposite directions: patients receiving abatacept experienced greater improvement in global disease activity than those receiving placebo (−8.0 ± 7.6 vs. −2.7 ± 6.7, *p* = 0.023); but physicians rated improvements to be greater in the placebo arm than the abatacept arm (−17.3 ± 23.2 vs. –11.9 ± 18.1, *p* = 0.048).

**Table 3:** Mixed models evaluating modified Rodnan skin score adjusted for disease duration

Effect	Estimate (95 % CI)	*p*-value
Disease duration	−0.9 (−1.4 to −0.4)	0.0046
Drug, abatacept vs. placebo	−9.8 (−16.7 to −3.0)	0.0114
Study visit	−0.1 (−1.2 to 0.9)	0.783
Study visit abatacept^a^	−0.9 (−2.2 to 0.4)	0.1773

^a^Refers to the mixed model taking into account the interaction between visit times and abatacept treatment. *CI* confidence interval

### Improvers from abatacept-treated group map to the inflammatory intrinsic subset

**Fig. 1:**
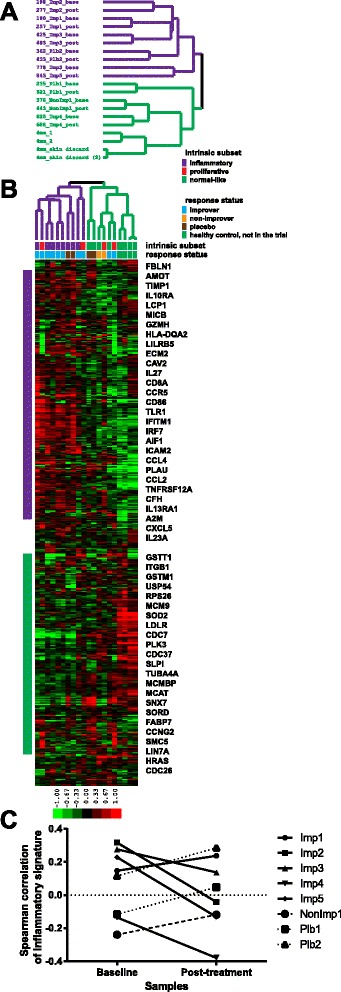
Intrinsic subset assignment. **a** Purple identifiers designate samples with increased expression of inflammatory gene signature and green identifiers correspond to samples with increased expression of normal-like gene signature. **b** Expression patterns of 645 intrinsic genes from [11] across samples from the study. ‘Intrinsic subset’ row shows results of formal intrinsic subset assignment using Spearman correlation statistics (see Methods). Color bar here and on subsequent figures refers to median-centered log2 fold change. **c** Changes in inflammatory gene signature between baseline and post-treatment. Improvers – *solid lines*, non-improver – *dashed line*, placebos – *dotted lines*

We used the 995 intrinsic probes from [11] (collapsed to unique genes) to perform unsupervised hierarchical clustering of the abatacept samples and four healthy controls previously analyzed on the same DNA microarray platform (Fig. 1a). We found that four out of five improvers and one placebo showed the increased expression of an inflammatory gene signature (e.g., TIMP1, IL27, TLR1 and AIF1). One improver, one placebo, one non-improver and four healthy controls showed the increased expression of a mostly normal-like gene signature (e.g., GSTM1, LDLR, MCAT and FABP7) (Fig. 1b). A fibroproliferative signature was only weakly observed in this small set of data. Additional file 1 contains the list of the intrinsic genes and their expression data across all abatacept and healthy control samples.

Intrinsic subset was formally assigned to each of the abatacept samples using non-parametric Spearman correlation statistics between each sample and intrinsic subset centroids calculated from [11] (see Methods). Overall, there was a high degree of similarity between the two methods of intrinsic subset assignment (Fig. 1b). Additional file 2 lists Spearman correlation statistics for all samples from Fig. 1.

We find that four out of five improvers were classified as inflammatory at baseline while one improver was classified as normal-like. Four of the five improvers that showed a clinical response also showed a significant decrease in their inflammatory gene signature from Milano et al. [11] post-treatment (Fig. 1c; *p* = 0.014, paired *t*-test) whereas the non-improver and the placebo-treated patients showed an increase in their inflammatory gene signature. The single non-improver was classified as normal-like. Amongst the six patients treated with abatacept, those whose baseline intrinsic subset was inflammatory showed a trend toward greater improvement in mRSS at 24 weeks compared with the normal-like group (−13.5 ± 3.1 vs. –4.5 ± 6.4, *p* = 0.067). The patients treated with placebo were assigned to the normal-like and inflammatory subsets.

### Abatacept decreases the immune response signature and CD28-dependent signaling in improvers post-treatment

**Fig. 2:**
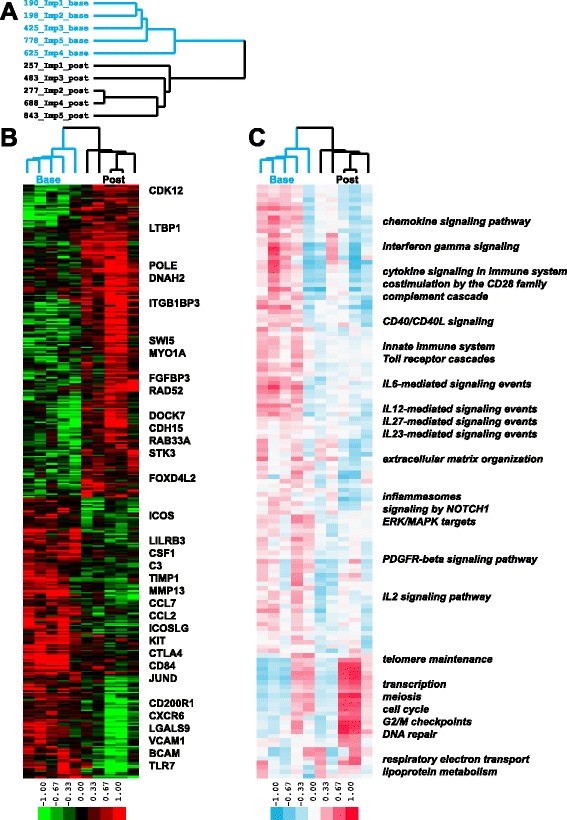
Gene and pathway signatures in abatacept improvers. **a** Blue identifiers designate baseline and black identifiers designate post-treatment samples; **b** 398 genes showed significant differential expression (*p* < 0.05) between baseline and post-treatment improver samples during the course of abatacept treatment; **c** 133 pathways were significantly differentially expressed in improvers (FDR <10 %). Color bar here and on subsequent figures represents single sample Gene Set Enrichment Analysis Normalized Enrichment Score (ssGSEA NES)

We identified 398 genes as significantly differentially expressed (*p* < 0.05, paired *t*-test) between baseline and post-treatment in the improvers (Fig. 2a). Genes with increased expression in improvers post-treatment included genes associated with general cell growth and cell cycle-related processes such as *DNA repair* (POLE, SWI5 and RAD52), *microtubule cytoskeleton* (DOCK7) and *mRNA processing* (CDK12). Genes with decreased expression in improvers post-treatment included genes associated with immune activation (e.g., *T cell proliferation*, *T cell costimulation*, *inflammasome*) and included chemokine ligands and receptors (CCL7, CCL2 and CXCR6), adhesion molecules (VCAM1, BCAM), T cell co-stimulator molecules (ICOS, ICOSLG), complement components (C3, C1S) and other genes known to play a role in the immune system (Fig. 2b). This subset of the improver gene signature (188 genes) was significantly enriched (FDR <5 %) in several immune system-related terms (e.g., *immune system process*, *immune response*, *cell activation* and *leukocyte aggregation*). The entire output for the 398 improver gene signature is listed in Additional file 3.

We investigated the expression of the improver gene signature across non-improver and placebo patients. Genes upregulated in improvers at baseline (including immune response genes) had significantly higher expression compared to non-improver and placebo baseline samples and to improvers post-treatment (*p* < 0.0001). Non-improver and placebo patients displayed stable expression of these genes (Additional file 4A). Genes downregulated in improvers at baseline (including cell cycle genes) had significantly lower expression compared to non-improver and placebo baseline samples and to improvers post-treatment (*p* < 0.0001). This trend was reversed in non-improver and placebo patients who displayed significant (*p* < 0.05) downregulation of those genes post-treatment (Additional file 4B).

We complemented the differential gene expression analysis with GSEA of the full set of genes in improvers before and after treatment. There were 106 pathways significantly downregulated in improvers post-treatment whereas 27 pathways were significantly upregulated in improvers post-treatment (FDR <10 %). Multiple immune response (e.g., chemokine and cytokine signaling) pathways went down and cell cycle functional terms went up in improvers post-treatment (Fig. 2c). The entire GSEA output is listed in Additional file 5.

**Fig. 3:**
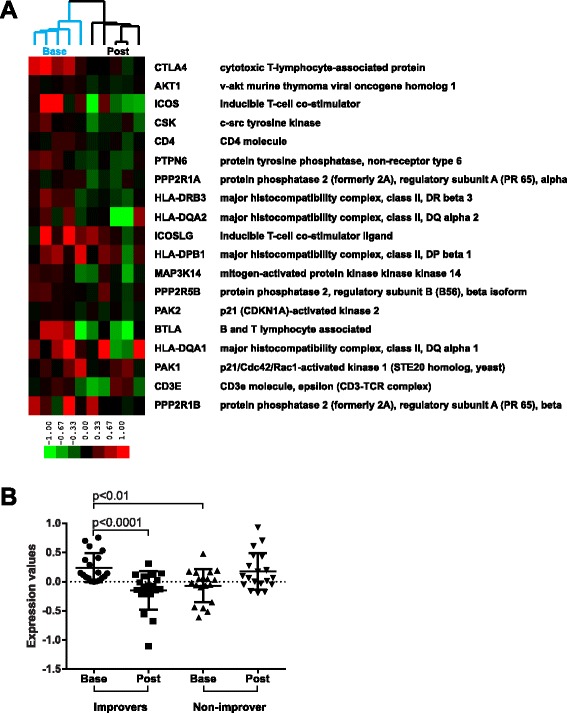
CD28 pathway trends across abatacept improver and non-improver samples. **a** Expression of 19 genes contributing the most to the GSEA enrichment score (core enrichment group) is shown in improvers. Genes are ordered by the GSEA rank metric score with those contributing the most to the enrichment score at the top and those contributing the least at the bottom. Array tree is from Fig. 1a. **b** CD28 pathway trends across improver and non-improver baseline (base) and post-treatment (post) samples. Expression values are for centroid vectors generated by averaging expression data for each of 19 genes across all respective samples (e.g., all improver bases). *p*-values are for paired (base vs. post) and unpaired (base vs. base) *t*-test comparisons. Graphs show mean with SD scatter plots

Since the mechanism of action of abatacept is to prevent the CD28-dependent co-stimulatory activation of T cells by binding to the CD80 and CD86 on antigen-presenting cells (APCs), we tested the hypothesis that we would observe a decrease in the expression of genes associated with T cell activation, specifically CD28-dependent signaling. We found that the Reactome molecular pathway, *costimulation by the CD28 family*, was significantly decreased post-treatment in abatacept improvers (FDR 7.7 %). This pathway comprises genes responsible for signaling events downstream of the CD28 superfamily of receptors that are required for the activation of T lymphocytes (in conjunction with the T cell receptor complex). Figure 3a shows 19 genes annotated to the CD28 pathway in Reactome that were the most significant contributors to the GSEA enrichment results. These genes were significantly upregulated in improvers versus non-improver at baseline (*p* < 0.01) and significantly downregulated in improvers post-treatment (*p* < 0.0001) suggesting the specific inhibition of CD28 co-stimulatory signals by abatacept therapy in improvers as opposed to the non-improver (Fig. 3b).

### Immune response pathways are increased at baseline in patients that improve with abatacept therapy

We found 14 pathways with significantly different baseline expression between improvers and a single non-improver via GSEA (FDR <10 %). Of 14 pathways, 13 were upregulated in improvers and included *complement pathway*, *integrin signaling* and *extracellular matrix organization* suggesting the enrichment in immune system functionality in abatacept improvers at baseline as opposed to the non-improver (Additional file 6B). Interestingly, the baseline sample tree based on the clustering of significant pathways was very similar to the baseline intrinsic subset assignment results from Fig. 1. Specifically, three out of four improvers classified as inflammatory at baseline clustered together, whereas a single non-improver and an improver, both classified normal-like, also clustered together (Additional file 6A). The fact that patient Imp4 clustered with a non-improver suggested that the former is likely to be a spontaneous improver.

### Placebo patients show increased lipid metabolism at baseline relative to abatacept-treated patients

**Fig. 4:**
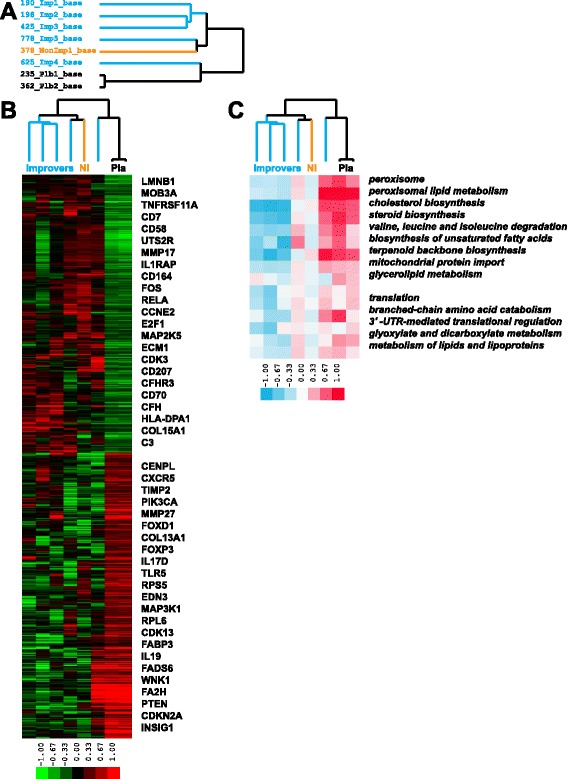
Gene and pathway signatures between abatacept and placebo groups at baseline. **a** Blue identifiers are improvers, black identifiers are placebos and orange identifier is non-improver; **b** 1,640 genes had significant differential expression at baseline between abatacept and placebo groups (*p* < 0.05); **c** 15 GSEA pathways were significantly differentially expressed at baseline between abatacept and placebo groups (FDR <10 %)

We identified 1,640 genes that were significantly differentially expressed (*p* < 0.05, unpaired *t*-test) at baseline between abatacept and placebo samples (Fig. 4a). Of the genes with increased expression in the abatacept group, 810 had a heterogeneous functional profile displaying the enrichment in both proliferative (*transcription from RNA polymerase II promoter*, *DNA binding*, *mitotic cell cycle*) and inflammatory (*innate immune system*) terms (FDR <5 %), and 830 genes that were highly expressed in the placebo group were enriched in functional terms associated with lipid metabolism (e.g., *lipid biosynthetic process*, *lipid metabolic process*, *sterol biosynthetic process*, *peroxisome*) (FDR <5 %) (Fig. 4b). All 15 significant pathways from GSEA procedure (FDR <10 %) were upregulated in the placebo group and were mostly related to lipid metabolism (Fig. 4c).

### Abatacept-treated patients show a decrease in inflammatory pathways

**Fig. 5:**
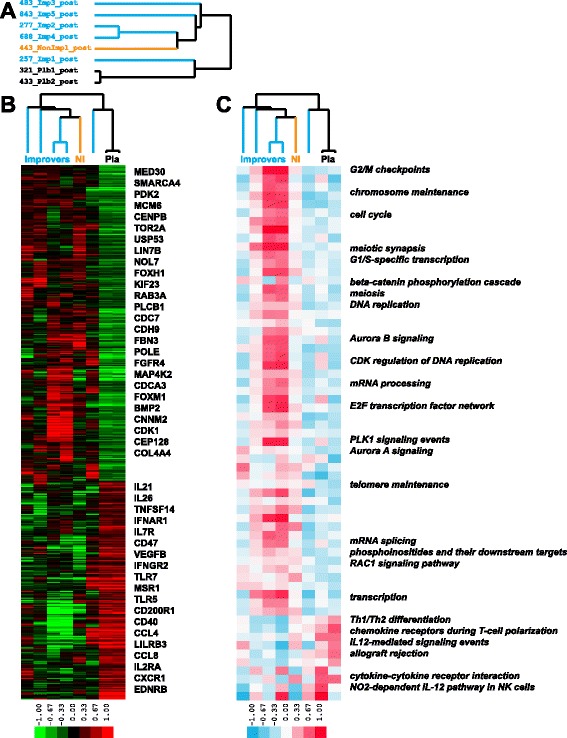
Gene and pathway signatures between abatacept and placebo groups post-treatment. **a** Blue identifiers are improvers, black identifiers are placebos and orange identifier is non-improver; **b** 1,354 genes were significantly differentially expressed between abatacept and placebo groups post-treatment (*p* < 0.05); **c** 63 pathways had significant differential expression between abatacept and placebo groups post-treatment (FDR <10 %)

We identified 1,354 genes significantly differentially expressed (*p* < 0.05, unpaired *t*-test) between abatacept and placebo patients post-treatment (Fig. 5a). Of the genes upregulated in abatacept post-treatment samples, 801 were enriched in *cell cycle*, *chromosome segregation* and *nuclear division* (FDR <5 %), while 553 genes with the increased expression in placebo post-treatment samples were enriched in *cell proliferation*, *inflammatory response*, *cytokine production* and *immune system process* (FDR <5 %) (Fig. 5b). GSEA identified 63 pathways with significant differential expression between abatacept and placebo samples (FDR <10 %); 53 pathways increased in the abatacept group post-treatment were related to cell cycle (e.g., *G2/M checkpoints*, *meiosis*, *DNA replication*, *Aurora B signaling* and *PLK1 signaling*) whereas 10 pathways upregulated in placebo post-treatment samples were related to the immune response (e.g., *Th1/Th2 differentiation*, *IL12 signaling* and *cytokine-cytokine receptor interaction*) (Fig. 5c). These results suggest that abatacept-treated patients lost inflammatory signature whereas placebo-treated patients became more inflammatory post-treatment.

We also compared GSEA results specifically between improvers from the abatacept group (Fig. 2c) and a single placebo-improver patient; 12 pathways were significantly differentially expressed in the latter (FDR <10 %). All of the pathways were down at baseline and subsequently increased over time in the placebo sample (Additional file 7). Five out of twelve pathways were in common with the pathways that were significantly decreased post-treatment in the abatacept-improver group and included *IL12 signaling*, *NOD-like receptor signaling* and *Toll-like receptor signaling* pathways. The remaining pathways were unique to the placebo-improver patient and included increased *CD8+ T cell signaling*, *NFkB activation* and *IL17 signaling* pathways. This analysis shows that the inflammatory signature increased post-treatment in the placebo-improver patient whereas it decreased post-treatment in the abatacept-improver group.

### CD28-dependent signaling is specifically decreased post-treatment in the abatacept group, but not in the placebo samples

**Fig. 6:**
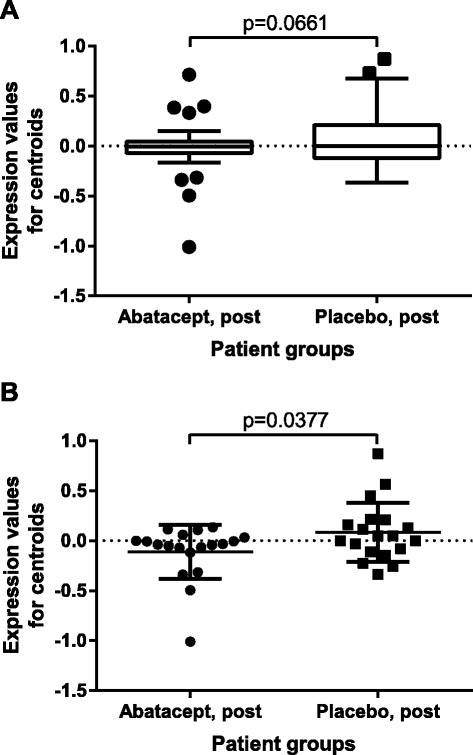
CD28 pathway trends across abatacept and placebo post-treatment groups. **a** Comparison of expression centroids for the entire set of genes annotated to CD28 pathway. *p*-value is for unpaired *t*-test with Welch’s correction. Graph is Tukey’s box and whiskers plot. **b** Comparison of expression centroids for the core enrichment subset of CD28 pathway from Fig. 3. *p*-value is for unpaired *t*-test. Graph shows mean with SD scatter plot

Finally, we analyzed the expression trends of the entire gene set annotated to the *costimulation by the CD28 family* pathway across abatacept and placebo post-treatment samples (62 genes). Abatacept samples displayed a trend towards lower expression of these genes post-treatment compared to placebos (*p* = 0.0661, unpaired *t*-test with Welch’s correction) (Fig. 6a). We then looked exclusively at the 19 genes that formed a core enrichment group of this pathway based on the GSEA results for improvers (Fig. 3a). The abatacept group had a significantly lower expression of this gene signature post-treatment compared to placebos (*p* = 0.0377, unpaired *t*-test) (Fig. 6b) suggesting that the gene expression changes of the relevant molecular pathway in the abatacept group were treatment-specific and did not occur in the placebo group.

## Discussion

Our study suggests that abatacept therapy is associated with distinct changes in gene expression that are primarily seen in those with a positive clinical response. Genes that were significantly differentially expressed in abatacept improvers between baseline and post-treatment either showed stable expression or displayed opposite trends in non-improver and placebo samples suggesting the association with a clinical response to abatacept treatment. The intrinsic subset assignment of abatacept patients shows that the improvers tend to be in the inflammatory intrinsic subset at baseline. The majority of the improvers demonstrate the loss of the inflammatory signature post-treatment consistent with the robust response to the therapy. These changes in the gene expression profiles of improvers are reflected by the functional changes as seen in the differentially regulated pathways. Generally, we observe decreased expression of genes involved in immune response, including those associated with CD28 T cell co-stimulation. We observe that improvers from the abatacept group show a decrease in the inflammatory gene signature from Milano et al. post-treatment with a concomitant decrease in inflammatory pathways, including significant repression of CD28-dependent signaling. We do not observe a decrease of the inflammatory gene signature or inflammatory pathways in the non-improver and placebo-treated patients.

Our results suggest that abatacept therapy may be most beneficial for patients from the inflammatory intrinsic SSc subset, a hypothesis that must be rigorously tested in a larger clinical trial. This finding is consistent with the idea that patients in the inflammatory intrinsic subset are likely to benefit from drugs specifically targeting inflammation [22]. Our results parallel those from a recent report showing that SSc patients who experienced a clinically significant improvement in mRSS while treated with mycophenolate mofetil (MMF) (CellCept, Roche, Basel, Switzerland) were classified as inflammatory at baseline [10].

While our study provides some support for the idea that blockade of T cell costimulation may be useful in the treatment of cutaneous fibrosis in SSc, the small number of patients precludes any conclusions regarding its clinical efficacy. After adjusting for disease duration, abatacept significantly improved mRSS by −9.8 points compared with placebo at 6 months, exceeding the minimal clinical important difference of 5.3 [23]. An observational study using The European League Against Rheumatism (EULAR) Scleroderma Trials and Research (EUSTAR) database also showed that abatacept was safe and well tolerated in 12 patients with SSc [24]. This study found that a mean of 11 months of abatacept treatment resulted in improvement in several measures of joint disease, including swollen and tender joint count as well as the 28-count Disease Activity Score. There was no significant change in mRSS after abatacept treatment, but only half of the patients had dcSSc and the mean baseline mRSS was only 5 [24].

Our study was limited by the small sample size and the relatively short treatment duration. Given that the natural history of dcSSc is stabilization of skin tightening over time [1], and T cell infiltrates in the skin are detected early in disease, most clinical trials of immunomodulatory therapies target patients with early (≤18 months to ≤5 years) disease [25, 26]. In our cohort, 80 % had ≤5 years disease duration; however, two of the three patients in the placebo group had long-standing disease of 11 years. Defining early disease as ≤18 months, three out of seven of the abatacept patients, but none of the placebo patients, were classified as early. Therefore, it is possible that the abatacept group was more likely to improve spontaneously than the placebo group. However, accounting for disease duration, abatacept-treated patients demonstrated significantly greater improvement in mRSS than placebo-treated patients (*p* = 0.0114). In addition, disease duration has only a mild negative correlation with change in mRSS (r = −0.27), and the best time for intervention is still unclear [26]. The abatacept group also had lower baseline mRSS and HAQ-DI scores suggesting that these patients may have been more likely to improve despite treatment. The discrepancy between patient and physician global score changes indicates that these VAS scales may not be reliable outcome measures. Finally, we did not assess for effects on joint disease in our study.

In terms of the gene expression analyses, the small sample size required the use of uncorrected *p*-values for the identification of gene signatures. We accounted for this limitation by performing corrections for multiple hypothesis testing in our functional enrichment and GSEA procedures.

Our study provides supportive data for a multi-center placebo-controlled clinical trial of subcutaneous abatacept in early dcSSc that is currently ongoing. This study includes pre- and post-treatment skin biopsies for gene expression analyses, and will provide more definitive data as to whether there is a role for abatacept in the treatment of cutaneous fibrosis in SSc patients, particularly those in the inflammatory intrinsic subset.

## Conclusions

We performed a placebo-controlled randomized trial of abatacept in patients with dcSSc. Most patients treated with abatacept experienced an improvement in mRSS post-treatment, and the majority of improvers mapped to the inflammatory intrinsic subset at baseline. Multiple immune response pathways including specific molecular targets of abatacept were significantly downregulated in improvers post-treatment but were unchanged in non-improver and placebo groups. Our results suggest that abatacept may be particularly beneficial for dcSSc patients from the inflammatory intrinsic gene expression subset and warrant further investigation in a larger clinical trial.

## Additional files

Additional file 1:
**Expression data for intrinsic genes across abatacept samples.** CDT (clustered data table) file with median-centered expression values for 645 intrinsic genes across samples from the study and four healthy controls. Gene symbols in the UID column have been color-coded to designate genes with increased expression in the inflammatory samples (purple cell color) and genes with increased expression in the normal-like samples (green cell color). Additional file 2:
**Spearman correlation statistics for intrinsic gene centroids.** Spearman correlation statistics for expression centroids for 645 intrinsic genes across abatacept samples. First worksheet lists correlation coefficients and second worksheet lists associated *p*-values. Additional file 3:
**Complete list of genes significantly (** ***p*** **< 0.05) changed in abatacept improvers.** Detailed information about the improver gene signature. ‘Upregulated In’ column has either ‘base’ (upregulated in baseline samples) or ‘post’ (upregulated in post-treatment samples) value. Gene symbols and gene names are from the Agilent 8x60K platform annotation file. ‘Fold Change’ column contains the fold change for each gene calculated according to the following rules: 1) if post Mean > base Mean, then Fold Change = post Mean – base Mean; 2) if base Mean > post Mean, then Fold Change = base Mean – post Mean. Additional file 4:
**Expression trends of the improver gene signature.** (A) Trends for genes significantly upregulated at baseline (base) and downregulated in post-treatment (post) improver samples. (B) Trends for genes significantly downregulated at baseline (base) and upregulated in post-treatment (post) improver samples. *p*-values are for unpaired t-tests with Welch’s correction for base vs. base and paired t-tests for base vs. post comparisons. Graphs are Tukey’s box and whiskers plots. Additional file 5:
**Complete list of pathways significantly (FDR <10 %) changed in abatacept improvers.** (A) Pathways upregulated in baseline and downregulated in post-treatment samples. (B) Pathways downregulated in baseline and upregulated in post-treatment samples. For each pathway, the following information is provided: pathway name, size (number of annotated genes), core enrichment (number of annotated genes that contribute most to the enrichment result), *p*-value and FDR q-value. Additional file 6:
**Baseline pathway analysis in abatacept improvers and non-improver.** (A) Sample dendrogram. Blue identifiers designate improvers and orange identifier designates the non-improver. Rectangles designate baseline intrinsic subset (IS) assignments from Fig. 1, with purple corresponding to inflammatory and green corresponding to normal-like subsets. (B) 14 pathways were significantly differentially expressed between improvers and the non-improver (NI) at baseline (FDR <10 %). Additional file 7:
**Comparison of the pathway signatures between the abatacept-improver group and a single placebo-improver.** Lists of significantly differentially expressed pathways from GSEA were compared for the abatacept and placebo improver(s). Blue and yellow circles correspond to pathways differentially expressed in the abatacept improvers (upregulated at baseline and post-treatment, respectively). Green circle corresponds to the pathways upregulated post-treatment in the placebo improver (no pathways were downregulated). Pathway lists represent five pathways in common between abatacept and placebo improver(s) and seven pathways unique to the placebo improver. Venn diagram was constructed using [27].

## Abbreviations

AE: Adverse event
APC: Antigen-presenting cell
dcSSc: Diffuse cutaneous systemic sclerosis
DLCO: Diffusing capacity of the lung for carbon monoxide
ESR: Erythrocyte sedimentation rate
EULAR: European League Against Rheumatism
EUSTAR: EULAR Scleroderma Trials and Research
FDR: False discovery rate
FVC: Forced vital capacity
GSEA: Gene Set Enrichment Analysis
HAQ-DI: Health Assessment Questionnaire-Disability Index
IL: Interleukin
IV: Intravenous
KEGG: Kyoto Encyclopedia of Genes and Genomes
MMF: Mycophenolate mofetil
mRSS: modified Rodnan skin score
SSc: Systemic sclerosis
ssGSEA: single-sample Gene Set Enrichment Analysis
VAS: Visual analogue scale
