# Supplementary figures and images for: Gene expression changes reflect clinical response in a placebo-controlled randomized trial of abatacept in patients with diffuse cutaneous systemic sclerosis
Source: Arthritis Res Ther. 2015 Jun 13;17(1):159. doi: 10.1186/s13075-015-0669-3 (PMC4487200; doi:10.1186/s13075-015-0669-3)

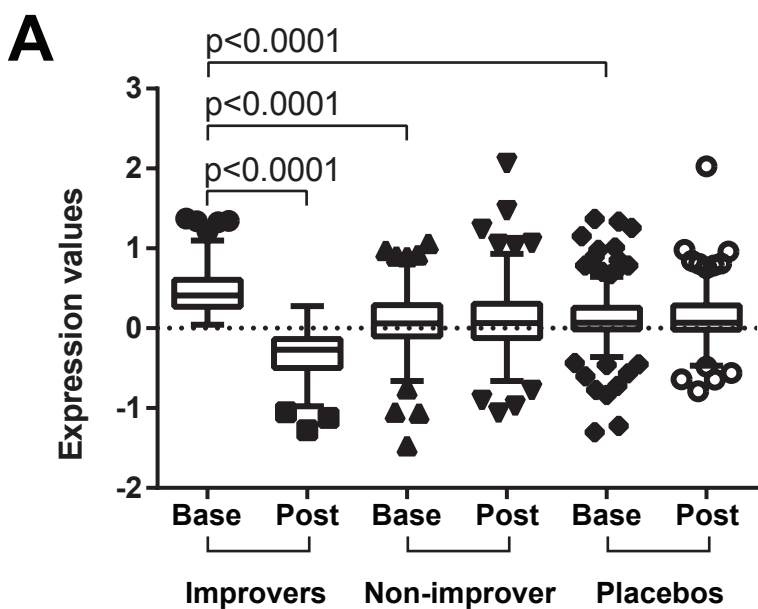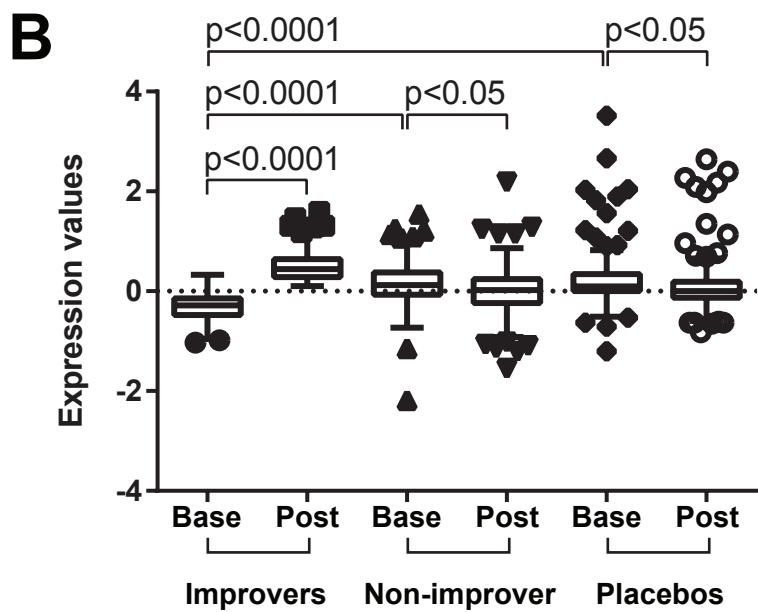

Supplement: Additional file 4: — Expression trends of the improver gene signature. (A) Trends for genes significantly upregulated at baseline (base) and downregulated in post-treatment (post) improver samples. (B) Trends for genes significantly downregulated at baseline (base) and upregulated in post-treatment (post) improver samples. p-values are for unpaired t-tests with Welch’s correction for base vs. base and paired t-tests for base vs. post comparisons. Graphs are Tukey’s box and whiskers plots. [file 13075_2015_669_MOESM4_ESM.pdf]

# A

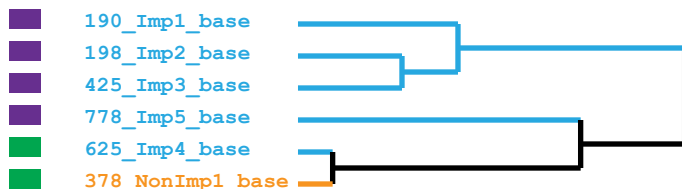

**Baseline  
intrinsic subset**

# B

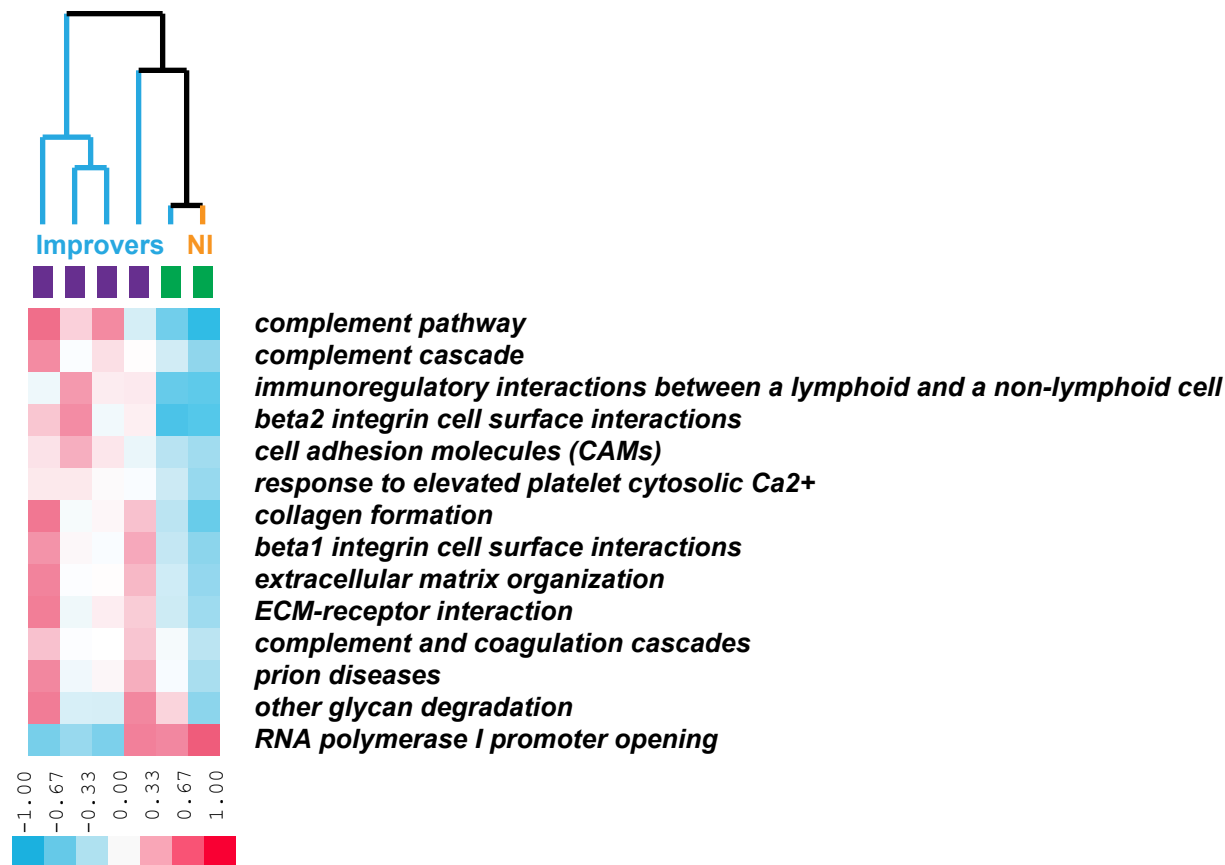

Supplement: Additional file 6: — Baseline pathway analysis in abatacept improvers and non-improver. (A) Sample dendrogram. Blue identifiers designate improvers and orange identifier designates the non-improver. Rectangles designate baseline intrinsic subset (IS) assignments from Fig. 1, with purple corresponding to inflammatory and green corresponding to normal-like subsets. (B) 14 pathways were significantly differentially expressed between improvers and the non-improver (NI) at baseline (FDR <10 %). [file 13075_2015_669_MOESM6_ESM.pdf]
