# Supplementary material for: Gene expression changes reflect clinical response in a placebo-controlled randomized trial of abatacept in patients with diffuse cutaneous systemic sclerosis
Source: Arthritis Res Ther. 2015 Jun 13;17(1):159. doi: 10.1186/s13075-015-0669-3 (PMC4487200; doi:10.1186/s13075-015-0669-3)
Supplement: Additional file 7: — Comparison of the pathway signatures between the abatacept-improver group and a single placebo-improver. Lists of significantly differentially expressed pathways from GSEA were compared for the abatacept and placebo improver(s). Blue and yellow circles correspond to pathways differentially expressed in the abatacept improvers (upregulated at baseline and post-treatment, respectively). Green circle corresponds to the pathways upregulated post-treatment in the placebo improver (no pathways were downregulated). Pathway lists represent five pathways in common between abatacept and placebo improver(s) and seven pathways unique to the placebo improver. Venn diagram was constructed using [27]. [file 13075_2015_669_MOESM7_ESM.pdf]

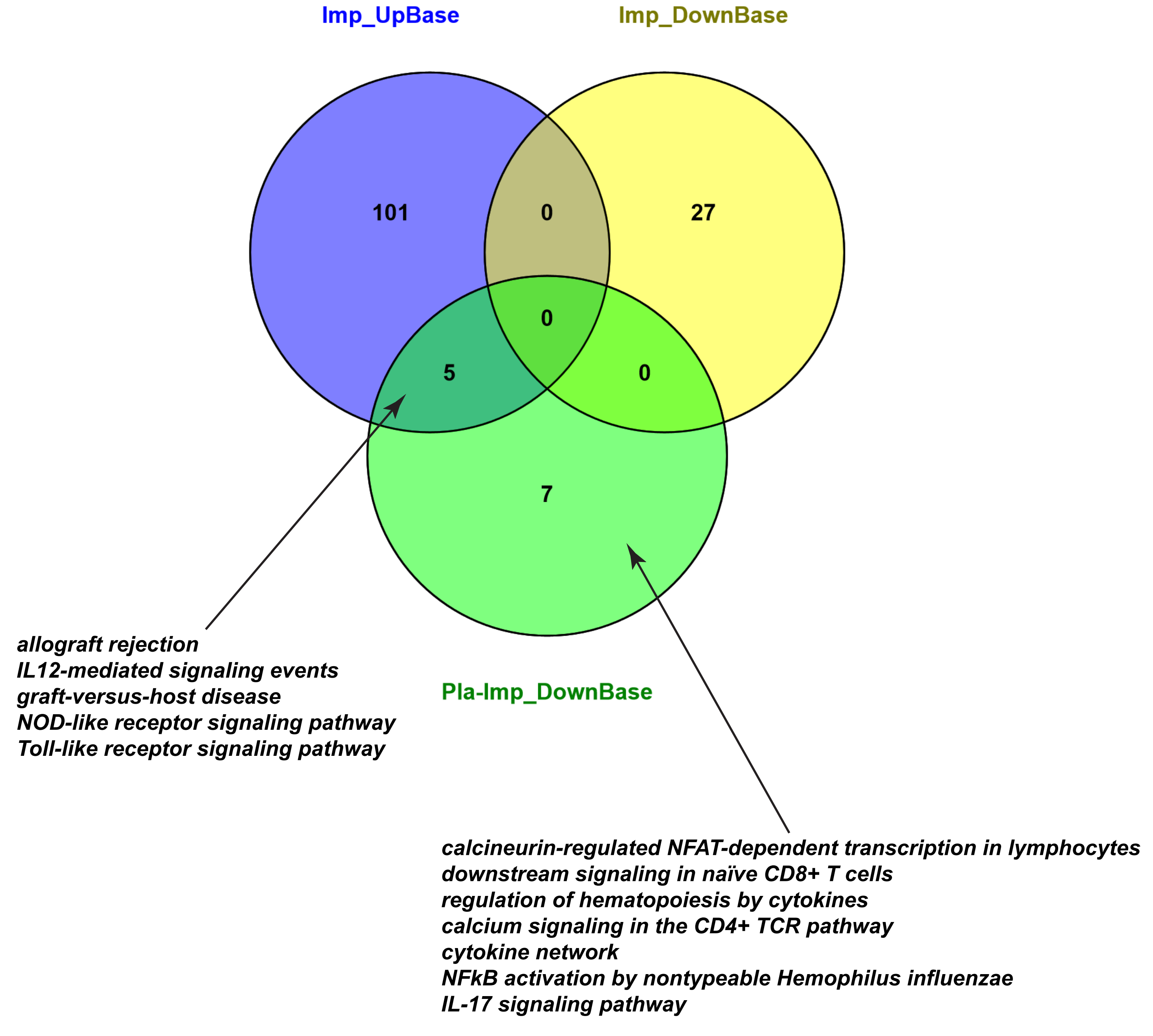
Imp_UpBase
Imp_DownBase
101
0
27
0
5
0
7
Pla-Imp_DownBase
allograft rejection
IL12-mediated signaling events
graft-versus-host disease
NOD-like receptor signaling pathway
Toll-like receptor signaling pathway
calcineurin-regulated NFAT-dependent transcription in lymphocytes
downstream signaling in naïve CD8+ T cells
regulation of hematopoiesis by cytokines
calcium signaling in the CD4+ TCR pathway
cytokine network
NFkB activation by nontypeable Hemophilus influenzae
IL-17 signaling pathway
